# Gut microbiome metabolism of plant phenolic glycosides and its relationship with human health

**DOI:** 10.20517/mrr.2026.17

**Published:** 2026-07-30

**Authors:** Clara G. de los Reyes-Gavilán, Nuria Salazar

**Affiliations:** ^1^Department of Microbiology and Biochemistry of Dairy Products, Instituto de Productos Lácteos de Asturias (IPLA-CSIC), Oviedo 33011, Spain.; ^2^Diet, Microbiota and Health Group, Instituto de Investigación Sanitaria del Principado de Asturias (ISPA), Oviedo 33011, Spain

## INTRODUCTION

Plants contain complex structural polysaccharides, while they also produce diverse secondary metabolites, such as terpenoids, alkaloids, and phenolic compounds, many of which are glycosylated. Glycosylation improves their solubility and contributes to regulating important functions such as protection from predators and microbial pathogens, plant development and endocrine functions, thus controlling plant-animal and plant-microbe interactions^[1]^. Phenolic glycosides display a high chemical diversity in their aglycone structure (monophenols to polyphenols), including different chemical classes (coumarins, flavonoids, non-flavonoids, *etc.*) linked to diverse carbohydrate moieties that could be monosaccharides, disaccharides, or oligosaccharides with diverse stereochemistry and linkage to the aglycone. Previous studies have demonstrated beneficial physiological functions associated with the consumption of plant glycosides^[2]^. Human metabolism of these compounds primarily focuses on deglycosylation reactions (mainly, but not limited to, O-glycosidic linkages) by gut epithelial cells, intestinal absorption, and biotransformation. In this regard, whereas phase II conjugation reactions extensively occur in enterocytes and the liver, hepatic phase I transformation reactions catalyzed by CYP450 enzymes are scarce^[3,4]^. The gut microbiome plays a central role in mediating metabolic transformation of plant glycans and glycosides. Polysaccharides are broken down into monosaccharides, whereas the glycosidic part of plant glycosides could be released from the aglycone by diverse microbial extracellular or cell wall/periplasmic enzymes hydrolyzing a variety of glycoside linkages^[3-5]^. Monosaccharides can enter the glycolytic pathway, providing energy for the microbiota^[5]^. Some released aglycones from phenolic glycosides can enter microbial cells by passive diffusion or active transport and undergo variable metabolic transformations depending on the microbiota composition and functionality, contributing to modulating their biological activity and host health effects^[4]^.

In spite of recent advances in the metabolism of dietary plant phenolic glycosides by the gut microbiome and their health effects, there is still a long way to go to precisely know the specific biochemical pathways and metabolic mechanisms involved, the microorganisms participating in such processes, and how and to which extent different microbiota/microbiome profiles and their intrinsic metabolic functionalities mediate the impact of phenolic glycosides in human health. 

## DIVERSITY IN UTILIZATION OF PHENOLIC GLYCOSIDES BY MEMBERS OF THE HUMAN GUT MICROBIOTA

**Figure 1:**
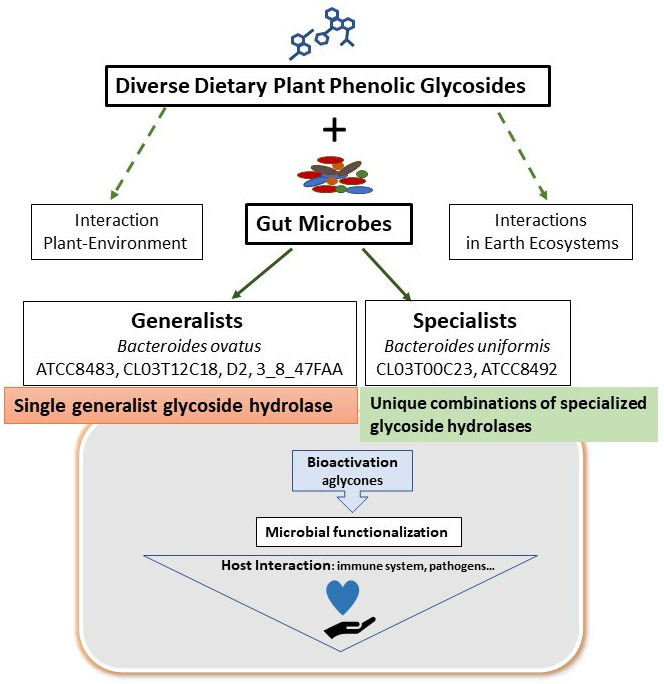
Graphical interpretation of the main findings by Kuziel *et al.* 2025^[6]^. Plants produce diverse phenolic glycosides. Some members of the human gut microbiota can encode either generalist single glycoside hydrolases that are able to metabolize some disaccharides and phenolic glycosides or combinations of specialized glycosyl hydrolases dedicated to specific phenolic glycosides. The functionalization of specific aglycones released by certain strains of *Bacteroides uniformis* mediates health effects in animal models.

A recent study by Kuziel *et al.*^[6]^ evidenced a high variation in the capacity to use for growth different plant glycosides among representative members of the human intestinal microbiota, encompassing from the use of all to none of the glycosides tested, and differing among different taxa or even among strains within the same species [Figure 1].

The authors demonstrated a high variation at the strain and aglycone-modification levels in the capacity to use glycosides within the genus *Bacteroides, *focusing on two clearly differentiated patterns of glycoside utilization. Thus, whereas generalist strains from *Bacteroides ovatus* are able to use a wide range of phenolic glycosides and disaccharides, some strains from *Bacteroides uniformis *specialize in specific glycosides, with comparatively higher growth yield in these compounds than in glucose. Using transposon-insertion mutagenesis coupled to next-generation sequencing (Tn-seq), genetic complementation, and chromatographic identification of released aglycones in single microbial cultures, the authors characterized the genetic and enzymatic basis of glycoside utilization in *Bacteroides*. Whereas *B. ovatus* harbors a single operon system containing a unique glycoside hydrolase (member of the GH16 family) to use a range of phenolic glycosides and disaccharides, *B. uniformis* uses different glycoside hydrolases from GH3 and GH16 families in unique combinations and located in different loci. Thus, the GH3 GshD hydrolase from *B. uniformis* demonstrated substrate specificity based on minor structural differences in the aglycone moiety of glycosides, whereas GH3 GshG and GH16 GGhC enabled the utilization of both aryl glycosides and disaccharides.

The order Bacteroidales is abundant in the human gut, *Bacteroides* being a highly prevalent genus. *Bacteroides* has a diverse metabolism, allowing these microorganisms to participate in the degradation of complex molecules, including polysaccharides and single sugars^[7]^. Notably, specialized hydrolases, such as those from specific *B. uniformis* strains are also present in plant and soil microorganisms specialized in hydrolyzing plant glycosides^[8]^ as well as in some food fermentative bacteria^[9],^ whereas the generalist pattern of glycosides and disaccharides utilization is common among members of human-associated microbiomes as *Bacteroides thetaiotaomicron*, *Enterococcus*, *Lactobacillus*, *Streptococcus *and *Escherichia coli*^[10,11]^*.* Jointly, findings from Kuziel and coworkers^[6]^ and from other authors suggest that generalist enzyme systems may have evolved primarily for sugar-sugar hydrolysis with broad or later expanded capacity to glycoside hydrolysis, whereas specialized systems may have evolved specifically to hydrolyze plant glycosides. 

## GUT MICROBIOTA MEDIATES INTESTINAL BIOACTIVATION AND FUNCTIONALIZATION OF PLANT PHENOLIC GLYCOSIDES

Kuziel *et al*.^[6]^ demonstrated particular biological functions mediated by microbiome bioactivation of specific phenolic glycosides [Figure 1]. Using *in vitro* models, the authors proved the *B. uniformis*-dependent conversion of polydatin into resveratrol and its antimicrobial activity against *Clostridioides difficile*, an opportunistic pathogen associated with gut microbiome recolonization in the setting of antibiotic use.

The authors found considerable diversity in inflammatory/anti-inflammatory *in vitro* potential in *B. uniformis*-released aglycones from plant glycosides. Aglycones such as saligenin, tyrosol, quercetin, and naringenin displayed anti-inflammatory profiles through the inhibition of TNFα and IL-6 cytokine production by lipopolysaccharide-stimulated macrophages, whereas the corresponding precursor glycosides did not. Remarkably, using a mouse model of intestinal inflammation, the authors demonstrated the role of the aglycone saligenin in protection of mice from colitis through the metabolism of *B. uniformis* on its precursor salicin, long associated with anti-inflammatory effects. 

## CHALLENGES DERIVED FROM THIS RESEARCH

This study demonstrates specific phenolic glycoside processing systems encoded by members of the human gut microbiota, supporting the generation of biological diversity of phenolic aglycone functions and raises important questions for future studies. 

-As suggested by the authors, it is of interest to characterize plant glycoside utilization systems and substrate specificity across different taxa from the human gut microbiota showing unique and differential patterns of phenolic glycoside utilization, as well as in microbial communities dedicated to plant processing (soil and silage ecosystems). Similar studies are interesting to be performed on members of the microbiome from herbivores, as well as humans subsisting mainly or exclusively on plant diets (vegetarians, vegans, culture-gathering farmers). It is also relevant to study microbial transformation of aglycones released from plant phenolic glycosides and whether/how this biotransformation could modify their bioactivity and mechanisms mediating their biological function in the host. 

-As human microbiota varies depending on health status, age, ethnicity, lifestyle, dietary habits, and medication, among others, some general questions arise: could these differential microbiota profiles affect host plant phenolic glycoside utilization? How could these potential alterations influence interactions of plant phenolic compounds with host functions, and how could they ultimately influence host health, medical treatments, prognosis and evolution of diseases, especially those affecting the digestive tract and immune system?

-Focusing on the establishment of microbiota in the infant gut, key related questions arise: could, and how, the type of feeding (breastfeeding/formula-feeding), weaning, and introduction of new foods affect the acquisition of phenolic glycoside utilization capacity by the gut microbiome? Considering the favorable effect on the intestinal immune homeostasis in mouse models of the microbiota-activated salicin into saligenin and the possibility that this also occurs with other aglycones and their precursor compounds, several hypotheses may be proposed about the ultimate influence that the acquired patterns of phenolic glycoside utilization by the microbiota may exert on allergy development, food intolerance and/or immunological diseases later in life. 

Kuziel *et al.*^[6]^ provide new insights into plant glycoside utilization by the gut microbiome, supporting further studies on the importance of feeding habits on these activities and their ultimate influence on human and global health. 

## DECLARATIONS

### Copyright

© The Author(s) 2026.
